# Target-based drug discovery through inversion of quantitative structure-drug-property relationships and molecular simulation: CA IX-sulphonamide complexes

**DOI:** 10.1080/14756366.2018.1511551

**Published:** 2018-09-17

**Authors:** Petar Žuvela, J. Jay Liu, Myunggi Yi, Paweł P. Pomastowski, Gulyaim Sagandykova, Mariusz Belka, Jonathan David, Tomasz Bączek, Krzysztof Szafrański, Beata Żołnowska, Jarosław Sławiński, Claudiu T. Supuran, Ming Wah Wong, Bogusław Buszewski

**Affiliations:** a Department of Chemistry, National University of Singapore, Singapore;; b Department of Environmental Chemistry and Bioanalytics, Faculty of Chemistry, Nicolaus Copernicus University, Toruń, Poland;; c Department of Chemical Engineering, Pukyong National University, Busan, Korea;; d Department of Biomedical Engineering, Pukyong National University, Busan, Korea;; e Interdisciplinary Centre for Modern Technologies, Nicolaus Copernicus University, Toruń, Poland;; f Department of Pharmaceutical Chemistry, Medical University of Gdańsk, Gdańsk, Poland;; g Department of Organic Chemistry, Medical University of Gdańsk, Gdańsk, Poland;; h Dipartimento di Chimica, Universita degli Studi di Firenze, Polo Scientifico, Laboratorio di Chimica Bioinorganica, Sesto Fiorentino (Florence), Italy;; i NEUROFARBA Department, Sezione di Scienze Farmaceutiche, Università degli Studi di Firenze, Sesto Fiorentino (Florence), Italy

**Keywords:** Drug discovery, inverse QSPR, molecular docking, molecular dynamics, carbonic anhydrases

## Abstract

In this work, a target-based drug screening method is proposed exploiting the synergy effect of ligand-based and structure-based computer-assisted drug design. The new method provides great flexibility in drug design and drug candidates with considerably lower risk in an efficient manner. As a model system, 45 sulphonamides (33 training, 12 testing ligands) in complex with carbonic anhydrase IX were used for development of quantitative structure-activity-lipophilicity (property)-relationships (QSPRs). For each ligand, nearly 5,000 molecular descriptors were calculated, while lipophilicity (log*k*_w_) and inhibitory activity (log*K*_i_) were used as drug properties. Genetic algorithm-partial least squares (GA-PLS) provided a QSPR model with high prediction capability employing only seven molecular descriptors. As a proof-of-concept, optimal drug structure was obtained by inverting the model with respect to reference drug properties. 3509 ligands were ranked accordingly. Top 10 ligands were further validated through molecular docking. Large-scale MD simulations were performed to test the stability of structures of selected ligands obtained through docking complemented with biophysical experiments.

## Graphical Abstract

**Figure:**
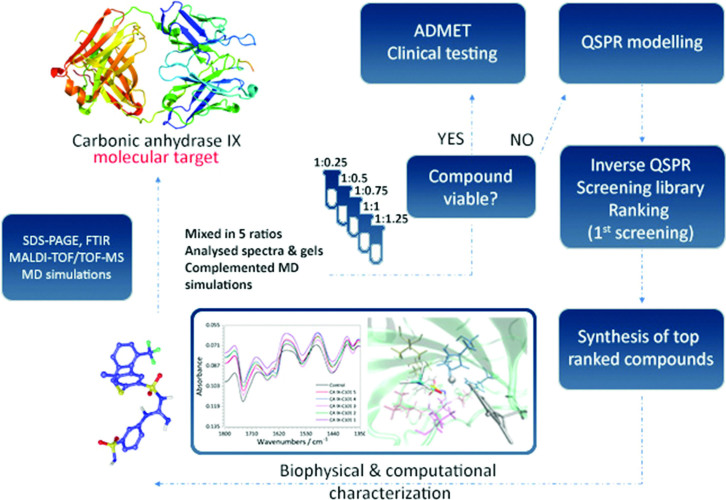

## 1. Introduction

Throughout the years, drugs were discovered by identification of their active ligands through observations in treatment or by occasional and serendipitous exploration. Drug design was dominated with structure-based methods, focussed on development and analysis of the ligands themselves. With the development of modern technologies, *in silico* methods such as target-based drug design (TBDD) and virtual ligand screening (VLS) have emerged as promising approaches for determination of active ligands for specific biological targets^1^. TBDD is a process which involves definition of a drug target which can be a gene, a genetic product^2^ or a compound related to a specific molecular mechanism^3^. Besides that, several bioassays and computational methods can be used to test molecular target-drug interactions in order to determine whether the drug results in activation or inhibition^4^. Modern genomic investigation has opened a door to discovery of numerous drug targets, which led to the development of entire libraries of ligands generated for molecular targets using computational drug design tools^5^.

Computational drug design tools include computer-aided drug design and discovery (CADD), ligand- and structure-based methods (incl. molecular docking, pharmacophore modelling), and afore-mentioned VLS. Structure- and ligand-based approaches greatly differ with respect to the information used for modelling. On top of that, 3 D structure of the target is not always known or troublesome to crystallise^6^. Molecular docking^7^ is a traditional method used in CADD in which the preferred orientation of a small molecule corresponding to its binding mode is *optimised* with respect to the target of interest resulting in formation of a stable complex. Docking algorithms can be applied for the search of potential ligands from a library, modelling of binding mode and affinity of candidate or known ligands^8^. In spite of efficiency of docking methods, pharmacophore modelling is used more frequently and generally requires less time^9^, although pharmacophore identification can on occasion arise from a docking study. It is also more precise than the traditional ligand-based approach^8^. However, protein flexibility is being recognised as of fundamental importance for wider applicability of docking methods and analysis of ligand-induced changes in protein binding sites. Simple molecular dynamics can be introduced for validation of structures obtained through molecular docking.

Disadvantages of all the mentioned methods can be improved by means of integration, i.e. integrated ligand- and structure-based approaches: (i) interaction-based and (ii) similarity-based docking. The former involves identification of interactions between the protein and target using known physico-chemical data, while the latter focuses on combination of structure-based docking methods with ligand similarity methods^10^ that makes VLS much more efficient^8^.

**Figure 1.**
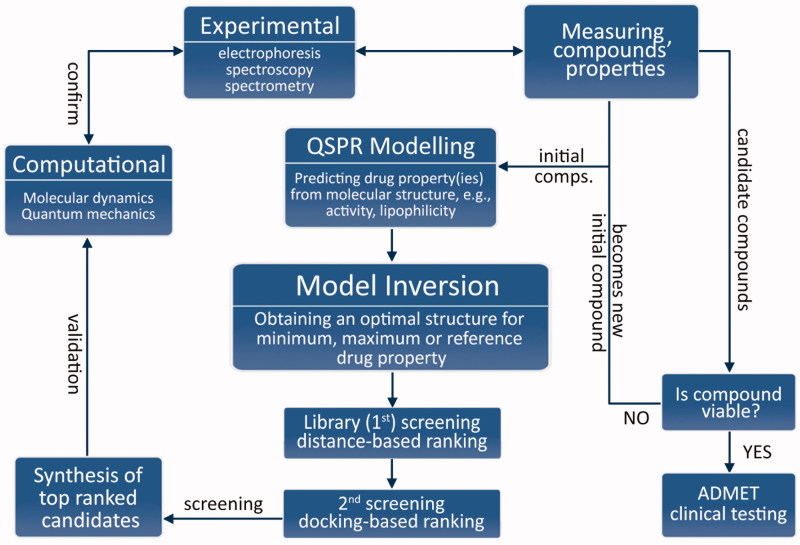
Flowchart of the proposed drug discovery methodology.

In this work, we tackle the problematics of integration in a different manner, with a synergistic methodology (Figure 1) combining experiments, high-throughput computing and mathematical programming, or mathematical optimisation. At its core, it follows the reasoning: *if drug properties are a function of molecular structure, then the ideal drug candidate’s molecular structure can be obtained as an inverse function of their desired value (e.g. minimum, maximum or with respect to a reference)*.

Upon validation of the model, it is inverted to obtain a molecular structure of an ideal candidate drug with desired properties (e.g. minimum or maximum y-values). This model inversion is essentially a constrained optimisation problem and should be done using an appropriate mathematical optimisation method because the relationship in Equation (1) is subjective, not injective (i.e. one-to-one) in general.

General optimisation formulation is:

(1) $$\begin{array}{c} x^{*}=\arg\;\min\;J(x)\\ s.t.\;\;\;h(x)=0;\;g(x)\leq 0\\ \;\;\;\;\;\;\;x_{i,\min}\leq x_{i}\leq x_{\max} \end{array}$$

This optimisation formulation of drug discovery gives great versatility of the proposed methodology because the objective function *J*(x) can be any form. It can be formulated as a minimisation/maximisation problem (e.g. minimising inhibition constant), or with respect to a reference. A solution of the inversion is screened against a ligand library (first screening) based on Euclidean distance of molecular descriptors and ranked for synthesis/evaluation. This can be done using search algorithms such as genetic algorithm when the library is large^11^. It is followed by a second screening using molecular docking.

Molecular docking and dynamics (MD) simulations are used to elucidate the interactions of the candidate ligands with the molecular target. After synthesis, biophysical experiments are performed to complement them, and vice-versa. Finally, the drug properties of the candidate drugs are experimentally determined. Thereby, the viability of candidate drugs for further testing is evaluated. If they are deemed not viable, they become new initial ligands, and the process is repeated.

Since synthesis can be time-consuming, and thereby out of the scope of our work, as a proof-of-concept we have focussed on the development of a drug property prediction model and its inversion, strengthened with computational and experimental structural studies: The model was inverted with respect to a reference value, with an aim to obtain a molecular structure of an ideal candidate drug. Carbonic anhydrase IX (CA IX) has been selected as a molecular target.

Regardless of the numerous experimental studies available on CA IX and its complexes, there is still only a few systematic MD models and simulations^12–15^. From a computational point of view, drug design methodologies combined with rigid-body docking and MD simulation are promising to investigate binding site(s), binding affinity and enzyme-ligand interactions^16–18^. Therefore, in this work, molecular docking studies were performed to additionally rank the ligands and validate the inverse QSPR. MD simulations were performed to evaluate the stability of the docked structures and elucidate non-covalent interactions between the ligands and the active site of CA IX. Biophysical experiments (SDS-PAGE, MALDI-TOF/TOF-MS, FTIR) were performed to complement the simulations.

## 2. Materials and methods

CA IX in complex with 45 sulphonamide-based inhibitors of previously determined purity^19^ ^,^ ^20^ (Table 1, Table S1) was used as a model system. Activity towards CA IX (expressed as log*K_i,_ pK_i_*) and lipophilicity (expressed as log*k_w_* ^21^) of the inhibitors were used as dependent variables to develop quantitative structure-activity-lipophilicity (drug property) relationships (QSPRs)^22^. Although not as popular in drug discovery as protein kinases,^23–26^ cancer-associated CA IX is still an interesting target due to its correlation with tumor cells proliferation^27–29^ In fact, a sulphonamide derivative selective to CA IX and CA XII developed in 2011 (SLC-0111) has recently entered phase II clinical trials for treatment of metastatic solid tumors^30^.

**Table 1.** Testing set ligands used for QSPR modeling and structural studies.

Ligands used for biophysical experiments are denoted in **bold**. Training ligands are shown in Table S1.

Both biophysical and computational experiments were performed on eight test ligands (Table 1) to elucidate the differences in ligands’ binding and in turn their activity towards CA IX. In all experiments, except to determine the inhibitory activity, recombinant CA IX was used (Sigma Aldrich, St. Louis, MO, USA).

### 2.1. Reversed-phase liquid chromatography (RP-LC) measurements

Stock solutions of the 45 ligands were prepared in 10 mM concentrations by dissolving them in dimethyl sulfoxide (Sigma Aldrich, St. Louis, MO, USA). Subsequently, they were diluted to 10 μM in a water: acetonitrile mixture (50:50% v/v). The acetonitrile used was MS-grade obtained from Merck (Darmstadt, Germany), while water was passed through the 18 MΩ Direct-Q 3 UV-R system (Merck, Darmstadt, Germany). Formic acid was purchased from Sigma Aldrich (St. Louis, MO, USA). RP-LC/MS experiments were performed using an Agilent 1260 system equipped with a UV-VIS spectrophotometric detector (Agilent Technologies, Santa Clara, CA, USA), an auto-sampler and a column thermostat. Optimal separation of the investigated sulphonamides was achieved using a Luna RP-C18 (4.60 × 150 mm, 3 µm, Phenomenex Inc., Torrance, CA, USA) column in linear gradient elution mode starting from 5% of phase A (water modified with 0.1% formic acid) to 95% of phase B (acetonitrile modified with 0.1% formic acid) with an injection volume of 10 μL. Two pairs of gradient chromatographic runs were performed: 0–30, and 0–60 min for less, as well as 0–40 to 0–60 min for more lipophilic ligands. Measurements were performed at 30 °C with an eluent flow rate of 0.3 ml min^−1^. Peak apex retention times obtained at these conditions were used to compute lipophilicity (expressed as log*k_w_*)^21^ using DryLab 2000 Plus software (LC Resources, Walnut Creek, CA, USA). To do so, the following equation was employed:

(2) $$\text{log}\;k=\;\text{log}\;k_{w}+S\varphi$$

where *k* represents the retention factor, S the linear slope, while φ represents the volume fraction of the organic modifier. Column, mobile phase, instrument conditions, and the elution program were taken into account to determine the parameters and coefficients of Equation (2).

### 2.2. Stopped-flow spectrophotometry

Stopped-flow spectrophotometry (Applied Photophysics (Oxford, UK) stopped-flow instrument) was used to assay the CA-catalysed CO_2_ hydration activity^31^. Phenol red in a concentration of 0.2 mM has been used as an indicator, at the absorbance maximum of 557 nm, with 10 mM Hepes (at pH 7.5) as buffer, and 0.1 M Na_2_SO_4_ (for constant ionic strength). The CA-catalysed CO_2_ hydration reaction was carried out in a period of 10–100 s. Concentrations of CO_2_ ranged from 1.7 to 17 mM for the determination of kinetic parameters and inhibition constants. Stock solutions of the inhibitors in a concentration of 1 mM were prepared in distilled-deionized water with 10–20% (v/v) of DMSO not inhibitory at these concentrations. The solutions were subsequently diluted up to 0.1 nM in distilled-deionized water. Inhibitor and enzyme solutions were pre-incubated together for 15 min at room temperature prior to the assay, to allow for the formation of the E–I complexes. Inhibition constants were obtained by non-linear least-squares fitting using PRISM 3 software (GraphPad Software Inc., La Jolla, CA, USA) as per refs.^32^ ^,^ ^33^ and represent the mean from three different determinations.

### 2.3. QSPR model development

Molecular structures of the 45 sulphonamides were drawn in ACD/Labs ChemSketch (Advanced Chemistry Development, Inc., Toronto, Ontario, Canada). First, a semi-empirical method AM1^34^ was used to pre-optimize the ligands, while Density Functional Theory (DFT)^35^ ^,^ ^36^ with the B3LYP^37^ functional at the 6–31 + G(d,p) level^38^ was used to refine the final geometries.

After that, an initial matrix of 4872 molecular descriptors was computed using Dragon 6.0 (Talete, Milano, Italy). Using three statistical pre-selection criteria: (i) critical relative standard deviation value (RSD) of 5%, (ii) critical pairwise correlation value of 0.8 (a descriptor with the lower correlation with the Y-variables was removed), and (iii) removal of all the descriptors with zero values, except binary and integer descriptors, it was reduced to 303 molecular descriptors. This 45 × 303 matrix was used as X-space (predictors), while experimentally-determined log*k_w_* and log*K_i_* were used as Y-space (dependent variables) for further modeling.

Subsequently, Kennard and Stone algorithm^39^ was used to uniformly separate the 45 samples into 33 training, and 12 validation set samples. Genetic algorithm coupled with Partial Least Squares (GA-PLS) as superior for variable selection^40^ ^,^ ^41^ was used in order to select a subset of molecular descriptors yielding the best QSPR model. This was achieved by minimising a multi-objective fitness function encompassing both root mean square error of estimation (RMSEE), and root mean square error of prediction (RMSEP):

(3) $$\eta =\sqrt{\eta_{1}{}^{2}+\eta_{2}{}^{2}}$$

where *η*_1_ and *η*_2_ represent:

(4) $$\eta_{1},\eta_{2}=\sqrt{\frac{\left(n_{T}-n_{S}-1\right)\text{RMSE}E_{1}{}^{2}+n_{P}\text{RMSE}P_{1}{}^{2}}{n_{T}+n_{P}-n_{S}-1}}$$

for log*k_w_* and log*K_i_* predictions, while *n*_T_, *n*_P_, and *n*_S_ represent the number of training samples, number of validation samples, and number of selected variables, respectively. Subscripts one and two represent log*k*_w_ and log*K*_i_, respectively. RMSEE and RMSEP are expressed using:

(5) $$\text{RMSEE},\text{RMSEP}=\sqrt{\frac{\sum_{i=1}^{n}{\left(y(\text{pred})-y(\text{obsd})\right)}^{2}}{n}}$$

Hyper-parameters and functions of GA were optimised as follows: cross-over fraction and mutation rate in [0.2:0.2:0.8], number of selected variables in [5:1:20], uniform, roulette and tournament selection functions, as well as scattered, single-point and two-point cross-over functions were tested. An optimal number of PLS latent variables was determined through 7-fold cross-validation.

The final GA-PLS model was validated through a validation set of ligands, and its applicability domain was defined. Cross-validation-analysis of variance (CV-ANOVA)^42^ was used for testing the model’s significance. RMSEP and mean relative error (MRE) values were reported for both drug properties:

(6) $$MRE=\frac{\sum_{i=1}^{n}\frac{\left|y\left(\text{pred}.\right)-\;y\left(\text{obsd}.\right)\right|}{y\left(\text{obsd}.\right)}}{n}$$

### 2.4. Library of CA IX inhibitors

The ChEMBL^43^ and BindingDB^44^ databases were screened for CA IX inhibitors and 4531 ligands were identified. Due to limited time, their structures were optimised using the AM1 semi-empirical only without DFT refinement. After geometry optimisation, seven molecular descriptors were calculated and out of 4531 ligands, only those that are structurally similar to the ligands used for training the QSPR model were selected for building our library (3497 including our own compounds, Table S3). Structural similarity was determined by computing the leverage values (*h*) of the ligands and removing all those with a value higher than the leverage threshold h*.

**Table 3.** Final ranking after validation through molecular docking.

Ligand ID	Dist(X)	Inverse QSPR ranking^a^	IFD Score/kcal mol^–1^	Validation ranking	Final ranking^b^
C101	0.044	1	–504.11	6	4
C108	0.044	2	–503.69	7	5
A1279	0.081	3	–495.03	12	8
A0504	0.097	4	–496.64	9	7
C102	0.099	5	–505.78	1	3
A0277	0.100	6	–503.22	8	7
C97	0.110	7	–504.76	4	6
A0337	0.115	8	–493.14	14	11
A0456	0.115	9	–504.90	3	6
C106	0.149	11 (26)	–505.40	2	7
C105	0.340	12 (233)	–504.29	5	9
C75	0.429	13 (315)	–493.66	13	13
C89	0.982	14 (541)	–495.48	11	13

^a^The number in parentheses represents ranking if all the library ligands with h* < 0.727 are considered, h* refers to critical leverage. (Table S4).

b Final ranking represents the average between the inverse QSPR ranking and ranking obtained through molecular docking.

### 2.5. Inverse QSPR

In order to obtain an optimal molecular structure of a candidate drug, methodology of Jaeckle and MacGregor^45^ was re-designed. Generally, through inversion^45^ of a latent variable (e.g. PLS^46^) model, the optimal structure can be obtained by either minimising or maximising the desired drug property. However, this requires synthesising the ligands, which could be time-consuming, and is, thereby out of the scope of this work. Therefore, as a proof-of-concept, the inverse QSPR solution was obtained relative to reference values of drug properties (log*k_w_* of 4.470 and log*K_i_* of 1.204). Optimisation formulation of PLS inversion has then the following form:

(7) x*=arg min(log k^w− log kw,ref)2+(log K^i− log Ki,ref)2             s.t. [log k^w, log K^i]=Ct^;  t^=Px*;                    t^TSt−1t≤c1; ‖Pt^−x*‖2≤c2

where x* represents the optimal solution (i.e. optimal values of molecular descriptors), t represents X-space PLS scores, P represents X-space PLS loadings, C represents Y**-**space PLS loadings, S represents the sample covariance matrix of t, while *c*_1–2_ are constraint constants.

GA^47^ was used to solve the mixed integer nonlinear programming problem (Equation (7)), and the obtained solution was screened against the test samples including the reference ligand, as well as the ligands from the built library of CA IX inhibitors, based on Euclidean distances.

Computational time required for the database screening after inverse QSPR was measured using in-built MATLAB function *cputime* for up to three million simulated ligands (descriptors simulated using a random number generator).

Most of the computations were performed in MATLAB 2017a (Mathworks, Sherborn, MA, USA), whereas the CV-ANOVA analysis was performed using Simca 14.1 (MKS Data Analytics, Umeå, Sweden).

### 2.5. Molecular dynamics (MD) and docking studies

In the context of TBDD, molecular docking is an *in silico* technique to predict the best binding mode (pose) of a small-molecule ligand to a target protein (receptor) of interest, given the three-dimensional structures of both. The revealed binding interactions could then explain the inhibitory or agonist functions of the ligand with respect to the receptor. In other words, docking predicts the structure of the resulting protein-ligand intermolecular, binary complex given the constituting structures, known binding site(s), and searching and scoring algorithms. In this way, essential interactions for binding could be identified, thus guiding subsequent drug design processes. This technique was particularly interesting because of the low computational cost it requires.

To begin with rigid-body docking study, the ligands (Table 1) were built in Maestro 11.1 (Schrödinger, LLC, New York, NY, 2017), followed by minimization and conformational search (systematic torsional sampling method) using OPLS3 force field using Macromodel 11.5 in Maestro suite. The protonation states of the ligands were corrected for the physiological pH using LigPrep module. Subsequently, the protein retrieved from RCSB PDB (PDB ID: **5FL4** ^48^) was first pre-processed using Protein Preparation Wizard in Maestro, including addition of hydrogens at given physiological pH. The grid of the active site of receptor was generated for rigid-body docking. All crystal waters were also removed.

The resulting docking poses were then examined further *via* Induced-Fit Docking (IFD) procedure in Maestro, where possible, to refine the result by allowing the sidechains within the active site to alter their positions. To further investigate the validity of these docking poses, MD experiments were performed, using the following procedures. The fully bonded model was used to preserve the tetrahedral geometry of the metal site centre of CA IX in which bond terms (i.e. bond stretching, angle bending, torsional) were parametrized^49^. Although several enhancements are available such as including polarisable bonds, orbital hybridisation, and ligand field stabilisation energy, these are not readily available for routine use in modelling of metalloprotein-ligand complexes^50^. Practically, overly sophisticated models can even lead to worse or invalid results^51^. The bonded model, therefore, provides a balance between performance and accuracy.

Standard Induced Fit Docking (IFD) procedure was used, utilising the IFDScore metric defined as:

(8) IFDScore=1.0×GlideScore+0.05×Prime Energy

where GlideScore approximates binding affinity of the ligand to the receptor, while Prime Energy approximates the energy of the receptor after being induced to fit the tested ligand.

X-Ray crystal structure of CA IX obtained from the RCSB Protein Data Bank (PDB) database with PDB ID: **5FL4** ^48^ was used as a basis for the MD model. It comprises 257 amino-acid residues, and it was determined using X-Ray crystallography at a resolution of 0.182 nm.

GROMACS 5.0.4^52^ software with the AMBER force field^53^ was used for MD simulations under periodic boundary conditions. Parameters of the Amber99SB*-ILDN^54^ were used for all atoms.

The ligands were parametrised using the generalised AMBER (GAFF) force field^55^, while the metal site center was parametrised using the workflow developed by Li and Merz Jr.^56^ All the parameters were derived based on QM calculations using DFT^35^ ^,^ ^36^ with the CAM-B3LYP^57^ functional at the 6–31++G(d,p) level^38^ of theory. Prior to the parameterisation procedure, the originally optimised geometries used for QSPR were all deprotonated since sulphonamides bind to CA IX as anions^58^, while hydrogens were added to the protein structure using the H++ server^59^.

For the MD simulations, eight CA IX-ligand complexes were modelled. Ligands were placed into the active site based on the experimental coordination of 5–(1-naphthalen-1-yl-1,2,3-triazol-4-yl)thiophene-2-sulfonamide (**9FK**) within CA IX^48^. First, the ligands were superimposed to the structure of **9FK** using the S-N atom pair of the sulphonamide group. Subsequently, the ligands were rotated around the N-C bond of **9FK** to resolve any possible clashes with the protein. The rotation angle differed case-by-case with respect to the ligands’ bulkiness.

Next, the complexes were solvated with TIP3P^60^ water molecules in a cubic box and electrostatically neutralised with either Na^+^ or Cl^–^ ions. Bad contacts and structural clashes were removed with 5000 steps of energy minimisation, after which the systems were heated to 298.15 K at a constant volume for 60 ps. Density was equilibrated by subjecting the complexes to constant pressure (1 bar) and temperature (298.15 K) conditions for 1.0 ns.

Positions of heavy atoms (except water and ions) were restrained with a harmonic constant of 1000 KJ mol^−1 ^nm^−2^ during energy minimisation, heating and equilibration. Production MD simulations were performed for 1 µs without any restraints. Non-bonded interactions were truncated with a 1 nm cutoff. Long-range electrostatic interactions were treated with the particle-mesh Ewald method^61^. Potential-shift^62^ was applied for modification of van der Waals interactions. Finally, trajectories of all simulations were saved every 10 ps for visualisation and analysis using Maestro and GROMACS tools. MD simulations were validated by comparing the simulated flexibility profiles with standardised crystallographic temperature factors (B-factors)^63^ computed from experimental data, as well as computing the radius of gyration.

### 2.6. Sodium dodecyl sulfate-polyacrylamide gel electrophoresis (SDS-PAGE)

Stock solutions of protein and inhibitors were prepared by dissolving appropriate amount in ultrapure water and DMSO, respectively. The dissolving of protein was followed by sonication, vortexing and, centrifugation. Protein and solutions of each inhibitor were mixed at different molar ratios as follows: 1:0.25; 1:0.5; 1:0.75; 1:1 and 1:1.25. 4 µL of protein stock solution, relevant volume of inhibitor stock solution corresponding to each ratio and water were mixed in 2-ml Eppendorf tube. 10 µL of lithium dodecyl sulfate (4X Bolt, Thermo Fisher Scientific, USA) sample buffer, 4 µL of reducing agent (10X Bolt) and 26 µL of previously prepared sample were thoroughly mixed in 2-ml Eppendorf tube, centrifuged for 1 min at 10,621 × g and incubated for 10 min at 70 °C. Gel electrophoresis procedure was finished after 30 min, the gel was opened and stained in the dye for 1 h using an orbital shaker, placed into ultrapure Milli-Q water for destaining for 24 h.

### 2.7. Matrix-assisted laser desorption ionisation-time-of-flight-mass spectrometry (MALDI-TOF/TOF-MS)

0.5 µL of the mixture prepared for SDS-PAGE measurements for each inhibitor was spotted to ground steel target (Bruker Daltonics, Germany) with saturated solution of α-Cyano-4-hydroxycinnamic acid as a matrix using dried droplet technique. Mass spectra were obtained using MALDI-TOF/TOF-MS (Bruker Daltonics, Bremen, Germany) equipped with a modified neodymium-doped yttrium aluminum garnet (Nd: YAG) laser operating at the wavelength of 355 nm and frequency of 2 kHz. Results were obtained in linear positive mode with the mass range of 15,000–60,000 (m/z) with a laser power of 60% and global attenuator offset of 50%. Recorded spectra were smoothed using weighted adjacent-averaging with periodic boundary condition.

### 2.8. Fourier-transform infra-red spectroscopic (FTIR)

To register new vibrations that could correspond to a formation of binding between potential inhibitors and protein, mixture of protein and each inhibitor stock was prepared with total volume of 40 µL. Stock solution of CA IX in a quantity of 20 µL, while the volume of inhibitor stock varied according to the molar ratios and water was added till the final volume. Prepared samples (2 µL) were spotted to assay free-membrane card, let to dry and inserted to Direct Detect Infra-red Spectrometer (Millipore Sigma, USA).

## 3. Results and discussion

### 3.1. Reversed-phase liquid chromatography (RP-LC) measurements

**Figure 2.**
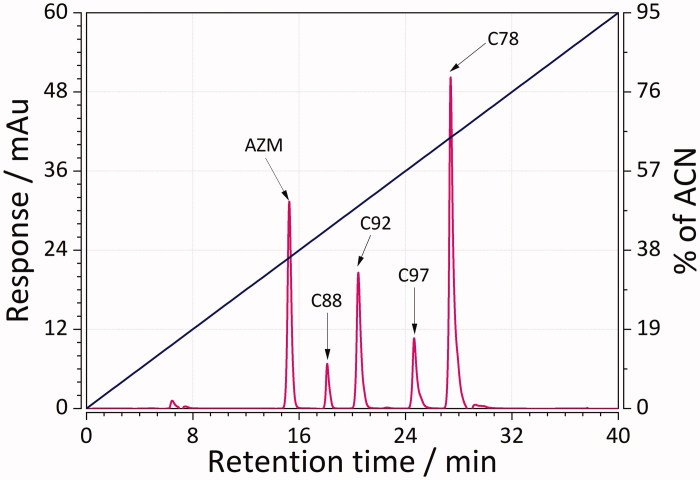
Representative mixture of five sulphonamides separated using RP-LC and a linear gradient from 0 to 95% acetonitrile in the mobile phase in a time of 0–40 min.

**Figure 3.**
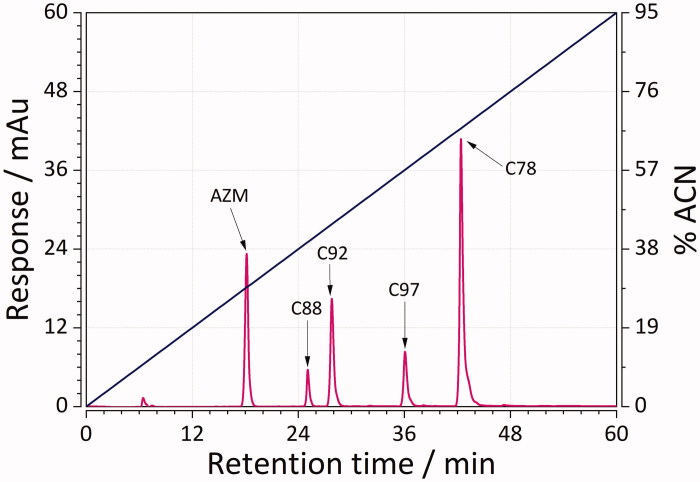
Representative mixture of five sulphonamides separated using RP-LC and a linear gradient from 0 to 95% acetonitrile in the mobile phase in a time of 0–60 min.

RP-LC was used to obtain (chromatographic) lipophilicity values of 45 sulphonamide ligands. This was achieved by performing two gradient chromatographic runs: 0–30, and 0–60 min for less, as well as 0–40 to 0–60 min for more lipophilic compounds. Representative chromatographic mixtures of five sulphonamides separated using RP-LC with short and long gradients are depicted in Figure 2 and Figure 3. Determined lipophilicity values (referred to as log*K_w_*, as defined in Equation (2)) ranged from 0.9 (weakly lipophilic) to more than 5 (strongly lipophilic ligands, Table S1). The results showed that the mixtures were well separated with good resolution in no more than 32 and 48 min for the case of short and long gradients. The elution order for both cases was as follows: **AZM** > **C88 **>** C92 **>** C97 **>** C78**. These results are, thereby, within our expectation because **AZM** has relatively more polar groups than nonpolar ones. Compound **C78**, on the other hand, is much more lipophilic due to the presence of fluorophenyl group.

### 3.2. Stopped-flow spectrophotometry

A library of 47 ligands tested with the CA-catalysed CO_2_ activity assay that have shown an excellent inhibition profile of CA IX have been utilized in this study. Most of them strongly inhibited the activity of this isozyme (K_i_ within the range of 2.8–651.6 nM; Table S1). It should be pointed out that 21 of the ligands exhibited higher or comparable activity compared to that of acetazolamide (K*_i_* = 25 nM), one of the most effective CA IX inhibitors approved for clinical use^64^. Despite its bulkiness, compound **C99** was reported to be the most potent (K*_i_* = 4.70 nM), about six times more potent than **AZM**. On the other hand, compound **C80** was the least potent (*K_i_* = 651.6 nM), being 26 times less potent than **AZM**. There was, however, no direct linear correlation between the reported log*K_i_* and log*K_w_* values, which is within our expectation. The significant features of this novel library leading to superior potency to that of **AZM** were, thereby, elucidated in the following sections.

### 3.3. QSPR model development

**Figure 4.**
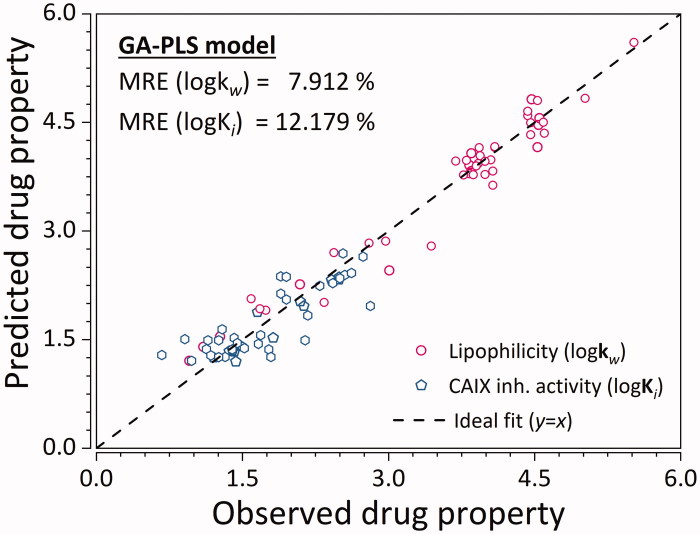
Predictive ability of the GA-PLS QSRR model for the training and testing set ligands. Empty hexagons denote log *K*_*i*_, while empty circles denote log*K*_*w*_. MRE denoted in the graph represents the sample mean value of MRE for training and testing ligands. (*n* = 45 × 2 = 90).

Upon optimisation of GA hyper-parameters, the model which gave the best ratio between model complexity and predictive ability was selected. Said model was comprised of seven molecular descriptors, and 4 latent variables. As such, it explained 87.30% variance of X-, and 77.12% of variance of Y-variables. Their respective testing set MRE values were 10.221 and 7.068% (Figure 4). Model has also shown to be statistically significant (Table S2) with *p*-values below the significance level of 0.05 for both y-variables.

**Table 2.** Solution of the PLS inversion and its distance to the molecular descriptor values of the reference ligand.

	IF-80	B05[O-S]	Sp_Min2,Bh_(i)	IVDE	G2e	H6e	B05[N–Cl]	Dist(X)
**X**	0	0	2.069	1.841	0.151	0.977	0	n.a.
X*	0	0	2.082	1.811	0.173	0.997	0	0.044
**ΔX**	0	0	0.013	0.030	0.022	0.020	0	n.a.

The solution of the PLS inversion is denoted by X*. All the abbreviations explained in the main text and/or Supporting Information.

The QSPR model was further tested using a library of 3497 CA IX inhibitors for AM1-optimised structures. The library was built from extensive screening of ChEMBL^43^ and BindingDB^44^ databases, and the CA IX inhibitors were screened based on structural similarity (Figure S1), with leverage values lower than the threshold 0.727 being retained. The large MRE (of ∼80%) arises from three main reasons: (i) the use of the semi-empirical AM1 method for optimisation instead of the DFT used in building the model, (ii) inherent empirical nature of the QSPR model, and (iii) several outliers (ligands for which the model is not trained for, i.e., not sulphonamides).

**Figure 5.**
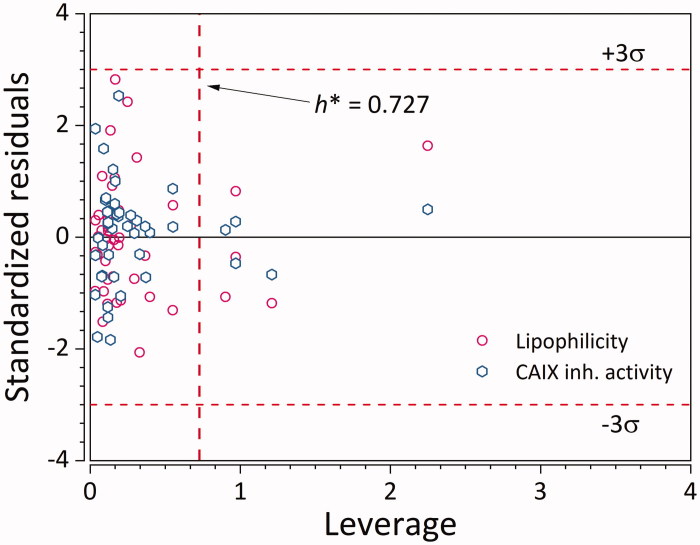
Applicability domain of the GA-PLS QSPR model for log*K*_*i*_. Empty hexagons denote log*K*_*i*_, while empty circles denote logkw. (*n* = 45 × 2 = 90).

Besides the use of a validation set, its applicability domain was also defined with three standard deviations of standardised residuals and critical leverage value of 0.727 as warning limits. Most of the ligands are within the applicability domain (Figure 5) for both Y-variables. Although there are several structurally distant ligands, their corresponding log*k_w_* and log*K_i_* values are very well predicted. This points to the fact that the developed GA-PLS model can accurately extrapolate log*k*_w_ and log*K*_i_ outside of the applicability domain.

Hotelling *T* ^2^ graph^65^ (Figure S2) confirms there are no outlying ligands. Although the graph depicts one possible outlier (outside of the critical *T* ^2^ value at the 99% confidence limit), it is expected that one out of 100 samples will be over it.

Therefore, the model is deemed robust, stable and validated. As such it was used for numerical inversion. The model consisted of seven molecular descriptors: (i) **IF-80**, (ii) **B05[O-S]**, (iii) **Sp_Min2,Bh_(i)**, (iv) **IVDE**, (v) **G_2e_**, (vi) **H_6e_**, (vii) **B05[N-Cl]**.

**Figure 6.**
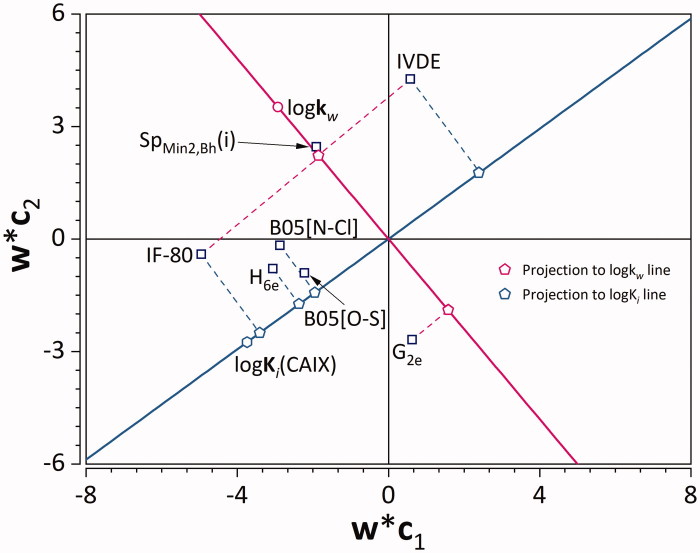
PLS loading plot. Empty squares denote X-, while empty circle and hexagon denote Y-variables. Projections to log*K_w_* and log*K_i_* lines are denoted using pink and light blue pentagons, respectively.

Multivariate correlation between these X-, and Y-variables was analyzed using the PLS loading plot (Figure 6) as in the work of Žuvela et al.^66^. Two lines were plotted to go through log*k_w_* and (0,0), as well as log*K_i_* and (0,0). The points representing **X**-variables were projected onto them. **IF-80** is a binary Ghose-Viswanadhan-Wendoloski anti-inflammatory drug-like index^67^. It corresponds to presence or absence of the ligand within a qualifying range (covering ∼80% of anti-inflammatory drugs): lipophilicity (expressed as ALOGP^68^) in a range of 1.4–4.5, molar refractivity in 59–119, molecular weight in 212–447 g mol^−1^, and number of atoms in 24–59. Apart from an obvious relation to lipophilicity, this descriptor is also associated with inhibitory activity of CA IX.

Hypoxia is entangled with inflammation on a cellular and molecular level wherein it can augment it^69^. It is not surprising then that some inhibitors of a hypoxia-inducible enzyme such as CA IX are classified as anti-inflammatory drugs, and that this descriptor explains part of the variance of Y-variables. This is evident from its strong positive correlation with lipophilicity, as it is far from the origin and in the same quadrant as log*k*_w_. (Figure 6). On the other hand, its strong positive correlation with log*K_i_* points to a decrease in inhibitory activity towards CA IX with the *true* value of **IF-80**.

**B05[O-S]** and **B05[N-Cl]** are topological binary descriptors that correspond to presence or absence of O-S, and N-Cl atom pairs at every five bonds, respectively. These two descriptors exhibit intermediate positive correlation with log*K_i_*, pointing to a decrease in inhibitory activity with their *true* value. From a chemical point of view, placing electronegative substituents, particularly halogen atoms in the immediate vicinity of each other, leads to a decrease in binding affinity towards CA IX.

**Sp_Min2,Bh_(i)** represents the second smallest eigenvalue in the Burden matrix weighted by ionisation potential. The Burden matrix^70^ is a weighted adjacency matrix with rows corresponding to atoms. The diagonal values of the matrix are atomic properties (e.g. ionisation potential), while the off-diagonal elements represent two bonded atoms (values range from 0.1, 0.2, 0.3 to 0.15 for single, double, triple, and aromatic bonds, respectively; 0.001 values if the atoms are not bound). Burden^70^ has observed a strong positive correlation between the eigenvalues of this matrix with lipophilicity expressed as log*P*. This is in accordance with the strong positive correlation of log*k_w_* with Sp_Min2,Bh_(i), observed on Figure 6 as it is far from the origin and in the same quadrant. There is no correlation with log*K_i_*.

**IVDE** is a topological descriptor derived from graph theory based on the partition of vertices according to degree equality^71^, and as such is a measure of molecular complexity. IVDE has exhibited a strong negative correlation to log*K_i_*, because its projection on the log*K_i_*-(0,0) line is in the opposite quadrant. Increase in IVDE values results in an increase of inhibitory activity towards CA IX (decrease of log*K_i_*). This points to an observation that more structurally homogenous and less bulky drug candidates more strongly inhibit CA IX.

**G_2e_**(second eigenvalue of the weighted covariance matrix weighted by Sanderson electronegativity) is a three-dimensional descriptor belonging to a family of Weighted Holistic Invariant Molecular descriptors (WHIM) descriptors^72^. It is a descriptor that encodes the symmetric spatial distribution of electronegativity with respect to invariant reference frames. It exhibited a medium negative correlation with lipophilicity. This means that symmetric distribution of electronegative atoms within the structure of the inhibitors leads to a decrease in their lipophilicity.

**H_6e_**represents the H-autocorrelation at a distance of six bonds weighted by Sanderson electronegativity^73^. It is based on constructing a leverage matrix (H) from three-dimensional molecular geometry for *i-j* atom pairs at a defined topological distance. Within the H-matrix, only atoms *j* at a distance of *d_ij_* bonds have a chance to interact with the *i*-th atom^73^. Its elements are multiplied by electronegativity values of individual atoms. This descriptor, thereby, encodes similar structural information as B05[N-Cl], and B05[O-S] descriptors, and it exhibits almost identical multivariate correlation towards log*K_i_* and log*k_w_*, only of a slightly higher magnitude.

### 3.4. Inverse QSPR

The developed and validated GA-PLS QSPR model was inverted through numerical optimisation using GA to minimise Equation (7). Since three molecular descriptors were binary/integer variables, mixed integer version of GA was used. Such GA has limitations on functions and hyper-parameters, so binary tournament selection function, adaptive feasible mutation function with a rate of 0.2, and a scattered cross-over function with a fraction of 0.8 were used. Critical DModX (α = 0.05) for the first constraint was 1.996, while critical Hotelling *T* ^2^ (α = 0.05) value for the second constraint was 11.923.

Inversion of the QSPR model resulted in values of molecular descriptors quite close to the reference values (Table 1).

The calculated value of Euclidean distance between the solution and the reference ligand was 0.044 (Table 2). The ligands were ranked based on Euclidean distance from the solution (Table 3), and molecular docking was used for validation. The discrepancy between the ranking obtained through docking and the ranking obtained through inverse QSPR is reasonable, even though the compound C101 is not #1 in the final ranking. The discrepancy can be attributed to the empirical nature of both the developed QSPR model and the scoring function used for docking. Employing molecular docking for analysis of protein-ligand interactions accounts only for binding, and strong binding may not necessarily correspond to strong inhibitory activity. The trend in the final ranking is retained when compared to the inverse QSPR one.

Based on simulated ligands using randomly generated molecular descriptors, it was found that the first round of screening for up to three million ligands is performed in real-time (Figure S3).

### 3.5. Sodium dodecyl sulphate polyacrylamide gel electrophoresis (SDS-PAGE)

SDS-PAGE allows for the observation of molecular masses of CA IX and its complexes. Two bands are present for the control sample (recombinant CA IX) in the electropherograms (Figure S3). The first band appears at the range of 44–45 kDa and the second at the range of 88–90 kDa. This is a consequence of the dimerisation process of CA IX in solution. Both the recombinant and the native form of CA IX exist as a mixture of monomeric and dimeric species in solution^74^. In electrophoretic analysis of CA IX-sulphonamide complexes, two bands were also observed for each concentration of inhibitors. However, with SDS-PAGE we were unable to able to obtain validated differences between points which represent different inhibitor concentrations.

### 3.6. Matrix-assisted laser desorption ionisation-time-of-flight-mass spectrometry (MALDI-TOF/TOF-MS)

For precise determination of molecular mass of CA IX and CA IX–inhibitor complexes, MALDI-TOF/TOF-MS was used. The measured molecular masses of recombinant CA IX (control) were 44482 ± 1 Da. Single-ionised [M-H]^+^ and double ionised [M-H]^2+^ CA IX and CA IX-inhibitor signals were registered confirming the dimerisation process. Unfortunately, we were unable to register resolved MALDI spectra for CA IX-C101, -C105, -C106, and -AZM, due to suppression of CA IX with sorbed inhibitors. MALDI analysis allowed for the differentiation of molecular masses between recombinant CA IX and its complexes. For instance, MALDI spectra point to the binding of one molecule of inhibitor C75, while two molecules of C84 inhibitor bind to CA IX (Figure S4). In case of the CA IX-C75 complex, one molecule binds to the active site of CA IX according to a well-known stoichiometric mechanism^75^. For the CA IX-C84 complex, one molecule of inhibitor is bound to the active site, while the second to a new binding site within the enzyme due to structure relaxation because of the sorption process taking place in solution.

In turn, it was shown that the structure of the inhibitors also has influence on the binding process. The influence of the secondary binding site on the inhibitory activity of the ligands has yet to be explored. Furthermore, due to the nature of the MALDI process (i.e., it is induced by laser), only strong molecular binding of inhibitors to the protein is registered^76^. The appearance of a second binding site could be an artefact of the analysis, due to the laser-induced binding mechanism. Weak hydrophilic/hydrophobic interactions can be observed using spectroscopic methods.

On the other hand, the drastic decrease in intensity for CA IX-inhibitor complexes when compared to the control points to signal suppression caused by sorption of the inhibitors themselves onto the enzyme, as in the case of C101, C105, C106, and AZM. Moreover, from the MALDI spectra (Figure S4) it can be observed that with the increase of protein-inhibitor molecular ratio, the maximum masses were reached mostly at the ratio 1:0.5. This phenomenon is a consequence of protein surface saturation by inhibitor molecules and establishment of the CA IX–inhibitors diffusion equilibrium^77^. It is also worth to point out that the larger ratios inhibitor: CA IX caused unstable bio-colloid systems^76^. Contrary to inhibitors 75 and 76, differences in masses of CA IX-inhibitor 80 complexes and control sample were too small to be considered validated. This was due to possible creation of sodium and/or water adducts.

### 3.7. Fourier-transform infra-red spectroscopic (FTIR)

The spectroscopic analysis in the infra-red range has confirmed interactions of all the inhibitors with the protein molecules. Changes in bands originating from amide I and II occur in case of all inhibitors (Figure S5) and indicate sorption processes onto the protein in solution. This sorption was caused by interactions of different nature between active functional groups of CA IX and inhibitors. Consequently, the protein molecules underwent relaxation processes that resulted in shifting and intensity change of amide bands. For complexes with acetazolamide and C106 (Figure S5), FTIR spectra exhibit shifting from 1443 to 1441 and 1442 cm^−1^, 1545 to 1542 and 1543 cm^−1^, 1660 to 1658 and 1659 cm^−1^. However, for concentrations 1 and 2, for the second vibration shifting from 1443 to 1444 cm^−1^ has been observed. New vibrations have been detected for all concentrations at 1631, 1632, 1633 and 1634 cm^−1^ (due to C=O and C=N stretching for Amide I band, Arginine residue)^78^.

In case of CA IX-76 complex (Figure S5), there is shifting of wavenumbers: 1374 to 1375, 1441 to 1442 cm^−1^ as well as shifting to lower frequencies from 1545 to 1543, 1660 to 1659 for all 5 inhibitor concentrations, 1738 to 1737 for only the second and fifth. In other words, for the second vibration (CN/CH group of Proline)^78^ the shifting to higher frequency was characteristic for all concentrations, while the opposite trend was observed for vibration second (N-H of Amide II band) and fifth (C=O of Amide I band).

**Figure 7.**
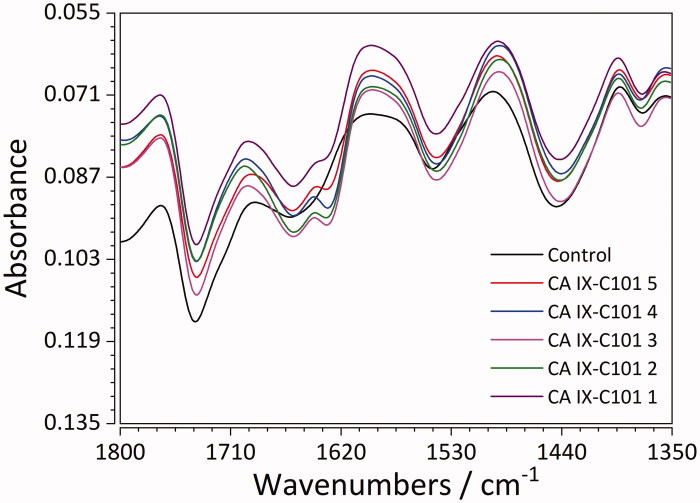
FTIR spectra of recombinant CA IX (control) and its complexes with the ligand C101 in 5 different concentrations.

Shifting to lower frequencies only for some concentrations (2, 5) was observed for the sixth vibration (pointing to C=O of Asparagine or Glutamine)^78^. These phenomena can be explained by hydrogen bonding, since the strength of original O-H bond is weakened when it becomes part of an O-H-N bond, as a result of interaction with functional groups of inhibitor 76. Moreover, specific shifting of band from 1545 to 1542 cm^−1^ (N-H of Amide II band) (e.g. CA IX – inhibitor 101, Figure 7) has shown hydrogen interaction between the amine and hydroxyl group of protein and inhibitors.^78^ For inhibitor 101, interactions with Arginine which resulted in C=N stretching of the Amide I band were a consequence of binding and relaxation processes.

Analysis of the FTIR spectra points to the fact that sorption of all inhibitors (for particular concentrations) on the surface of protein was caused by hydrogen bonding between amine and hydroxyl groups of CA IX and inhibitors as well as hydrophobic interactions between non-polar groups of inhibitor and aromatic amino acids of CA IX.

### 3.8. Molecular docking and MD simulations

**Figure 8.**
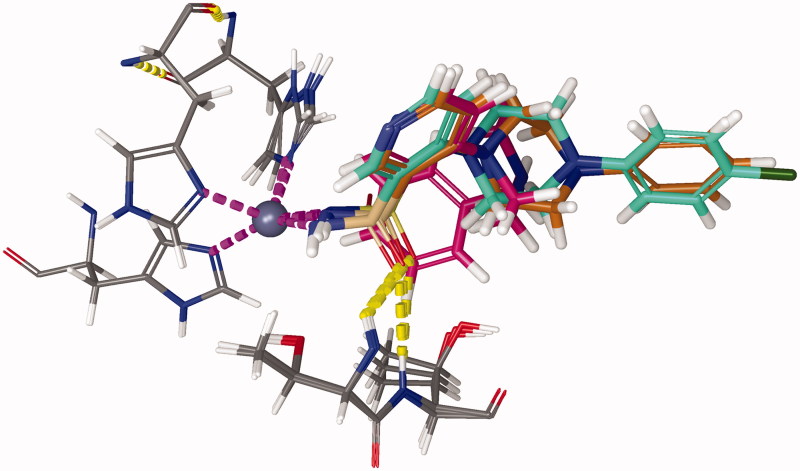
Superposition of binding poses of C75 (orange), C76 (faded teal), and C84 (salmon pink) against CA IX receptor (purple). Yellow dashed lines represent hydrogen bonds.

Molecular docking studies resulted in an (alternative) ranking to validate the inverse QSPR. For that purpose, standard Induced Fit Docking (IFD) procedure was used, utilising the IFDScore metric. Interaction analysis of the test ligands was performed for the top docking poses (Figure 8). For ligands C75, C76, C84, C105, and C106, one of the oxygen atoms of the sulphonamide moiety formed hydrogen bonds with backbone nitrogen atoms of THR200 and THR201, which could be perceived as oxyanion hole. The benzene ring of the benzene-sulphonamide moiety for the first three ligands, upon superposition, was retained faithfully.

For ligand C89, the hydrogen bond between oxygen atom of sulphonamide moiety and backbone nitrogen atom of THR200 was also predicted, but the hydrogen bond with THR201 was formed with the nitrogen of pyridine ring of C89. For ligand C101, the sulphonamide whose nitrogen was bound to zinc formed hydrogen bond with backbone nitrogen atom of THR200, and the secondary sulphonamide formed hydrogen bond with amido moiety of GLN71 sidechain. For ligand C105, halogen bond between the chlorine atom of the ligand and the nitrogen backbone atom of VAL130 was observed. For ligand C106, the secondary sulphonamide moiety formed hydrogen bond with amido moiety GLN92 sidechain. Upon docking, a final ranking was obtained (Table 3). Although there are discrepancies between the two rankings, they can be attributed to the error in the QSPR model, and the error of the scoring function used for molecular docking.

Large-scale molecular dynamics simulations were performed in order to confirm the stability of the docked structures. In this study, 1 μs long simulations were performed on CA IX complexes involving AZM, C75, C76, C89, and C101 ligands.

**Figure 9.**
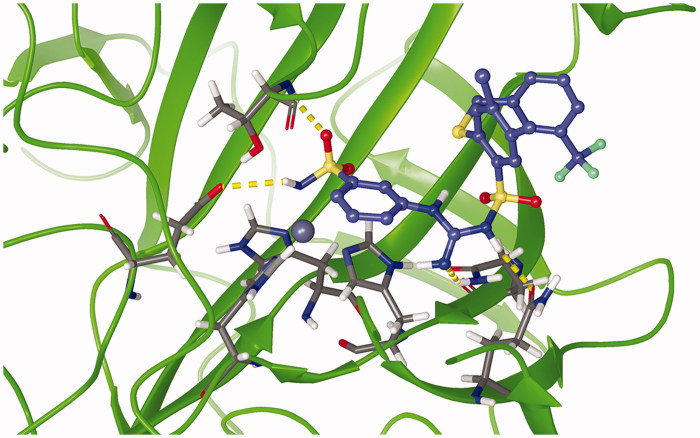
Representative snapshot of the structure of carbonic anhydrase IX (5FL4) in complex with C101 after 500 ns of MD simulation. Protein: green ribbon, zinc: silver sphere, C101: blue carbons, and 3 histidine residues: grey carbons. Magenta dashed lines represent the Zn-N covalent coordination bonds. Yellow dashed bonds represent hydrogen bonds.

Figure 9 depicts an energy minimised, equilibrated structure of CA IX-C101 complex after 500 ns of production MD simulation. It can be observed that the tetrahedral coordination between the zinc, three histidine residues and the ligand is conserved. Root mean square deviation (RMSD) was used as a measure of protein(-ligand) stability and convergence, with reference to C-α atoms of the respective energy-minimized protein-ligand structure It was observed that the RMSD values of C-α atoms in CA IX protein, in complex with AZM, C75, and C101 ligands, varied within range of 0.10–0.15 nm, whereas that with respect to CA IX-C76 complex varied within range of 0.15–0.20 nm.

Similar trend was also observed in CA IX-C89 complex with RMSD range of 0.15–0.20 nm for the first 500 ns of the simulation, then the value went up to a range of 0.35–0.40 nm for the rest of the simulation. To further examine the stability of the zinc prosthetic group in the active site, the RMSD values of this group upon least square fit of the C-α atoms were calculated as well.

The RMSD values varied within 0.10–0.15 nm for complexes involving AZM and C75 ligands, whereas for CA IX-C76 and C101 complexes, the RMSD value ranges were 0.20–0.25 nm and 0.35–0.40 nm, respectively. Similar trend to that in RMSD values of C-α atoms in CA IX-C89 complex was observed in RMSD values of the prosthetic group, where the values of the RMSD of the latter fell within range of 0.10–0.15 nm for the first 500 ns, then increased to 0.20–0.25 nm for the rest of the simulation. Thus, these results confirmed the stability of the simulations.

**Figure 10.**
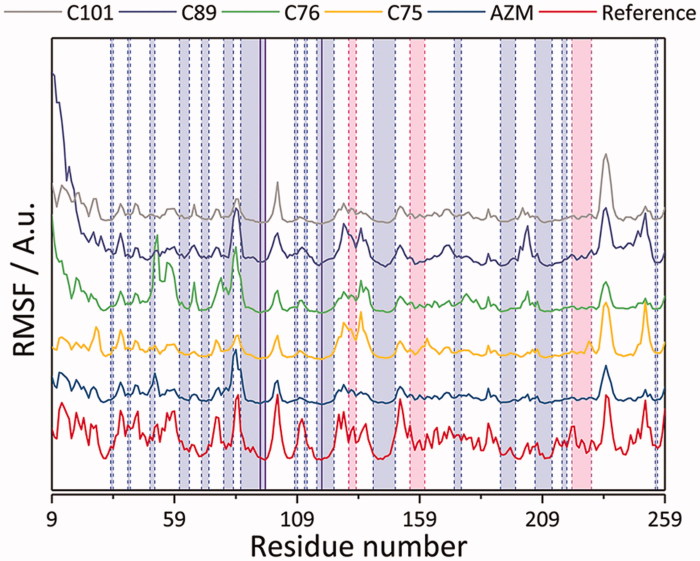
Root mean square fluctuations (RMSF) of studied CA IX complexes with AZM, C75, C76, C89, and C01 ligands. The reference values were calculated based on the crystallographic temperature factor (B-values)^63^ of PDB structure 5FL4.^48^ The light blue, pink, and white regions demarcated by the dashed blue and pink vertical lines correspond to β-sheet, α-helix, and loop secondary structures, respectively. The solid blue vertical lines correspond to the residues constituting the zinc prosthetic group: His 94, 96, and 119.

The simulations were further validated by comparing the root mean square fluctuation (RMSF), thereby standard deviation, trend to the experimental crystallographic temperature factor profile (B-values). It was found that the RMSF trend from the simulations follows the experimental one quite well (Figure 10). Further treatment of average structure examination, however, was taken for the case of CA IX – C101 and CA IX – C89 complex. Observing the dynamics of prosthetic group of the latter complex with respect to the average structure instead, the RMSD values were stable at around 0.10 nm upon fitting of C-α atoms.

For the former complex, the RMSD values of the zinc prosthetic group after fitting with respect to the C-α atoms and the average structure were stable at around 0.40 nm and 0.05–0.10 nm, respectively. This implies that the ligand was stable throughout the 1 μs long simulation, but it differed from the energy-minimized structure.

Furthermore, the hydrogen bond patterns formed between the protein active site and the studied ligands were examined, as shown in Table 4. In this paper, the hydrogen bond is defined as short-range contact between -OH/-NH donor moieties and O/N acceptor atoms where the distance cutoff between the two electronegative atoms is 0.35 nm, and the hydrogen-donor-acceptor angle cutoff is 30°. These distance and angle plots pertaining to the hydrogen bond can be found in Supporting Information.

**Table 4.** Summary of prominent hydrogen bonds formed throughout the 1 μs MD course.

Protein-ligand complex	Hydrogen bond (donor-hydrogen-acceptor)	Percentage of occurrence
CA IX – AZM (ligand ID: AM1253)	THR200N-THR200H-AM1253O2	64 %
	AM1253N3-AM1253H1-THR200OG1	41 %
CA IX – C75 (ligand ID: S51253)	S51253N1-S51253H23-THR200OG1	99 %
	THR200N-THR200H-S51253O4	83 %
CA IX – C76 (ligand ID: S61253)	S61253N1-S61253H23-S61253N11 (intramolecular, involving tertiary amine)	∼100 %
CA IX – C89 (ligand ID: C91253)	ARG244NH2-ARG244HH21-C91253O15	12 %
	C91253N16-C91253H28-GLU106OE1	18 %
CA IX – C101 (ligand ID: S11253)	S11253N28-S11253H51-THR200OG1	94 %
	THR200N-THR200H-S11253O30	72 %
	S11253N19-S11253H46-GLN71OE1	59 %

From Table 4, two kinds of relatively prominent hydrogen bonds in common were observed to engage THR200 (N or OG1) and THR201 (N). This agreed with the fact that THR200 acts as “doorkeeper residue”^79^, and this also agreed with the docking results mentioned earlier. In addition, hydrogen bond engaging GLN71 in CA IX – C101 complex which was predicted by docking results was also reproduced in the MD analysis (59% occurrence, Table 4).

## 4. Conclusions

In conclusion, a novel synergistic target-based approach for drug discovery combining experiments and high-throughput computing *in silico* techniques has been presented. The methodology is flexible, as it can be used for minimisation, and maximisation of the desired property, or with respect to a structure of an ideal drug candidate. The process begins by (i) performing experiments to determine the desired drug properties. It is followed by (ii) construction of a multivariate QSPR model. The developed model is (iii) inverted through mathematical optimisation and the solution is screened against a database of drug candidates (1st screening). In the meantime, (iv) molecular docking is performed to rank the compounds according to their binding energies (2nd screening). To test the stability of the structures of potential drug candidates, (v) molecular dynamics (MD) simulations are performed complemented with biophysical experiments. Finally, (vi) their respective drug properties are measured and if a compound is deemed acceptable, it proceeds to further testing. Otherwise, it becomes a new initial compound for QSPR modelling and the process repeats.

Since synthesising the candidate ligands is time-consuming, as a proof-of-concept the methodology was tested with respect to reference drug property values (those of ligand C101) with a strong inhibitory activity towards CA IX, as well as high lipophilicity.

A solution of the QSPR model inversion had values very close to the molecular descriptor values of C101. Based on the inverse QSPR results the ligands were ranked, which was validated through molecular docking. The first round of screening (after inverse QSPR) was performed in real-time (<1 s). Further testing of the QSPR model was realized using an external set with 3497 CA IX inhibitors. Furthermore, the developed QSPR model itself can also be used as a rapid and accurate tool for prediction of sulphonamides’ lipophilicity and activity towards CA IX.

To test the stability of the docked structures, MD simulations were performed. The MD study agreed with the claim that hydrogen bond formation with THR200 is essential towards binding affinity of the small-molecule inhibitors. Results of MD simulations were complemented with biophysical experiments.

Use of the fully bonded model for large-scale MD simulations has shown to be useful for qualitative and fragment-based semi-quantitative analysis of non-covalent interactions between sulphonamide inhibitors and CA IX. As such, it can be applied to other CA isozymes and their complexes which is particularly promising for the development of selective inhibitors.

## Supplementary Material

Supplemental Material
