# Supplementary material for: Target-based drug discovery through inversion of quantitative structure-drug-property relationships and molecular simulation: CA IX-sulphonamide complexes
Source: J Enzyme Inhib Med Chem. 2018 Sep 17;33(1):1430–43. doi: 10.1080/14756366.2018.1511551 (PMC6151961; doi:10.1080/14756366.2018.1511551)
Supplement: Supplemental Material [file IENZ_A_1511551_SM9007.zip › IENZ_1511551_Supplementary Material/2018-Zuvela_et_al-JEIMC_Supplementary_Information-08-09.doc]

Target-based drug discovery through inversion of quantitative structure-drug-property relationships and molecular simulation

Supplementary Information

Petar Žuvela^1,2^, J. Jay Liu^3,^*, Myunggi Yi^4^, Paweł P. Pomastowski^2^, Gulyaim Sagandykova^5^, Mariusz Belka^6^, Jonathan David^1^, Tomasz Bączek^6^, Krzysztof Szafrański^7^, Beata Żołnowska^7^, Jarosław Sławiński^7^, Claudiu T. Supuran^8,9^, Ming Wah Wong^1^, Bogusław Buszewski^2,5^

^1^Department of Chemistry, National University of Singapore, 3 Science Drive 3, Singapore, 11754, Singapore

^2^Department of Environmental Chemistry and Bioanalytics, Faculty of Chemistry, Nicolaus Copernicus University, Wileńska 4, 87-100 Toruń, Poland

^3^Department of Chemical Engineering, Pukyong National University, 365 Sinseon-ro, Nam-gu, 48-547, Busan, Korea

^4^Department of Biomedical Engineering, Pukyong National University, 45 Yongso-ro, Nam-gu, 48-513, Busan, Korea

^5^Interdisciplinary Centre for Modern Technologies, Nicolaus Copernicus University, Wileńska 4, 87-100 Toruń, Poland

^6^Department of Pharmaceutical Chemistry, Medical University of Gdańsk, Hallera 107, 80-416, Gdańsk, Poland

^7^Department of Organic Chemistry, Medical University of Gdańsk, Hallera 107, 80-416, Gdańsk, Poland

^8^Dipartimento di Chimica, Universita degli Studi di Firenze, Polo Scientifico, Laboratorio di Chimica Bioinorganica, Rm. 188, Via della Lastruccia 3, 50019 Sesto Fiorentino (Florence), Italy

^9^Università degli Studi di Firenze, NEUROFARBA Dept., Sezione di Scienze Farmaceutiche, 50019 Sesto Fiorentino (Florence), Italy

*Fax: +82 51 6296 429. E-mail: [jayliu@pknu.ac.kr](mailto:jayliu@pknu.ac.kr).

Contents:

**Table S1.** Experimental values of lipophilicities and activities towards CA IX for 45 investigated sulphonamide compounds.

**Table S2.** CV-ANOVA results for the **2-y**-GA-PLS model fit to 33 training compounds (α = 0.05).

**Figure S1.** Williams plot depicting leverage values of the CA IX inhibitors from the library. Only those with leverage values below the threshold (*h**) 0.727 were retained.

**Figure S2.** Hotelling T^2^ plot for the **2-y**-GA-PLS model; for 33 training compounds.

**Figure S3.** Plot depicting number of simulated compounds against CPU time.

**Figure S4.** SDS-PAGE gel electrophoresis bands. **A)** CA IX control (2 bands), marker solution (1 band), and CA IX-AZM complexes (9-13 bands), **B)** CA IX-C75, **C)** CA IX-C76, **D)** CA IX-C84, **E)** CA IX-C89, **F)** CA IX-C101, **G)** CA IX-C105, **H)** CA IX-C106.

**Figure S5.** MALDI-TOF-MS/MS spectra. **A)** CA IX-AZM, **B)** CA IX-C75, **C)** CA IX-C76, **D)** CA IX-C84.

**Figure S6.** FTIR spectra. **A)** CA IX-AZM, **B)** CA IX-C75, **C)** CA IX-C76, **D)** CA IX-C84, **E)** CA IX-C89, **F)** CA IX-C101, **G)** CA IX-C105, **H)** CA IX-C106.

**Figure S7.** Root mean square deviation (RMSD) for CA IX – AZM complex. Fitting with respect to the C-α atoms for CA IX, and the metal site centre.

**Figure S8.** Root mean square deviation (RMSD) for CA IX – C75 complex. Fitting with respect to the C-α atoms for CA IX, and the metal site centre.

**Figure S9.** Root mean square deviation (RMSD) for CA IX – C76 complex. Fitting with respect to the C-α atoms for CA IX, and the metal site centre.

**Figure S10.** Root mean square deviation (RMSD) for CA IX – C89 complex. Fitting with respect to the C-α atoms for CA IX, and the metal site centre.

**Figure S11.** Root mean square deviation (RMSD) for CA IX – C101 complex. Fitting with respect to the C-α atoms for CA IX, and the metal site centre.

**Figure S13.** Distance and angle plots (intermolecular hydrogen bond examination) for CAIX - AZM complex (legend; red: THR200N-THR200H-AM1253O2, olive green: AM1253N3-AM1253H1-THR200OG1).

**Figure S14.** Distance and angle plots (intermolecular hydrogen bond examination) for CAIX – C75 complex (legend; red: S51253N1-S51253H23-THR200OG1, olive green: THR200N-THR200H-S51253O4).

**Figure S15.** Distance and angle plots (intermolecular hydrogen bond examination) for CAIX – C76 complex (legend; red: S61253N1-S61253H23-S61253N11).

**Figure S16.** Distance and angle plots (intermolecular hydrogen bond examination) for CAIX – C89 complex (legend; red: ARG244NH2-ARG244HH21-C91253O15, olive green: C91253N16-C91253H28-GLU106OE1).

**Figure S17.** Distance and angle plots (intermolecular hydrogen bond examination) for CAIX – C101 complex (legend; red: S11253N28-S11253H51-THR200OG1, olive green: THR200N-THR200H-S11253O30, royal blue: S11253N19-S11253H46-GLN71OE1).

**Table S3.** Screening of 3464 CA IX inhibitors from ChEMBL and BindingDB databases for ones that are within the applicability domain of the developed QSPR model. Predicted and observed *pK*_i_, calculated values of seven molecular descriptors, and calculated values of standardized residuals and leverage values.

**Table S4.** Ranking of CA IX inhibitors that are within the applicability domain of the developed QSPR model based on the results of inverse QSPR.

**Tables S3** and **S4** are in a separate Excel file each in its own sheet.

Experimental values of lipophilicity and activity towards CA IX for 45 investigated sulphonamide compounds, and their molecular structures are listed in **Table S1**.

**Table S1. Experimental values of lipophilicity and activity towards CA IX for 45 investigated sulphonamide compounds and their molecular structures.**

| # | ID | Structure | | | | logk*_w_* | logK*_i_*(CA IX) |
| --- | --- | --- | --- | --- | --- | --- | --- |
| *1* | *AZM* |  | | | | *0.955* | *1.398* |
|  | | | | | | | |
| # | ID | R^1^ | R^2^ | R^3^ | | logk*_w_* | logK*_i_*(CA IX) |
| *2* | *C75* |  |  |  | | *3.010* | *2.093* |
| *3* | *C76* |  |  |  | | *4.530* | *2.130* |
| **4** | **C78** |  |  |  | | **3.440** | **2.170** |
| **5** | **C79** |  |  |  | | **5.520** | **2.533** |
| **6** | **C80** |  |  |  | | **2.970** | **2.814** |
| **7** | **C81** |  |  |  | | **5.020** | **2.739** |
| **8** | **C83** |  |  |  | | **1.740** | **1.894** |
| *9* | *C84* |  |  |  | | *1.270* | *1.654* |
| **10** | **C85** |  |  |  | | **1.590** | **1.951** |
| **11** | **C88** |  | **Me** | **H** | | **1.680** | **1.394** |
| *12* | *C89* |  | *Me* | *Me* | | *2.090* | *1.364* |
| **13** | **C90** |  | **Me** | **n-C_4_H_9_** | | **2.800** | **1.687** |
| **14** | **C91** |  | **Me** | **CH_2_CH_2_CO_2_Et** | | **2.440** | **1.290** |
| **15** | **C92** |  | **Et** | **H** | | **2.340** | **1.501** |
| *16* | *C93* |  |  |  | | *1.100* | *1.816* |
|  | | | | | | | |
| # | ID | R^1^ |  | | X |  |  |
| **17** | **C94** |  |  | |  | **4.050** | **1.415** |
| **18** | **C95** |  |  | |  | **3.930** | **1.146** |
| **19** | **C96** |  |  | |  | **4.090** | **2.143** |
| **20** | **C97** |  |  | |  | **4.090** | **0.908** |
| **21** | **C98** |  |  | |  | **3.920** | **1.130** |
| **22** | **C99** |  |  | |  | **4.070** | **0.672** |
| **23** | **C100** |  |  | |  | **4.530** | **1.519** |
| *24* | *C101* |  |  | |  | *4.470* | *1.204* |
| **25** | **C102** |  |  | |  | **4.430** | **1.771** |
| **26** | **C103** |  |  | |  | **4.460** | **1.322** |
| **27** | **C104** |  |  | |  | **4.600** | **0.978** |
| *28* | *C105* |  |  | |  | *4.550* | *1.431* |
| *29* | *C106* |  |  | |  | *4.540* | *1.415* |
| **30** | **C107** |  |  | |  | **4.430** | **1.792** |
| **31** | **C108** |  |  | |  | **4.460** | **1.176** |
| **32** | **C109** |  |  | |  | **4.590** | **1.255** |
| **33** | **C111** |  |  | |  | **3.900** | **1.447** |
| **34** | **C112** |  |  | |  | **3.940** | **1.663** |
| *35* | *C113* |  |  | |  | *3.830* | *1.477* |
| **36** | **C114** |  |  | |  | **3.990** | **1.255** |
| **37** | **C115** |  |  | |  | **3.830** | **1.892** |
| **38** | **C116** |  |  | |  | **3.800** | **1.949** |
| **39** | **C117** |  |  | |  | **3.870** | **2.550** |
| **40** | **C118** |  |  | |  | **3.770** | **2.428** |
| *41* | *C119* |  |  | |  | *3.850* | *2.491* |
| **42** | **C120** |  |  | |  | **4.070** | **2.619** |
| **43** | **C121** |  |  | |  | **3.990** | **2.294** |
| **44** | **C122** |  |  | |  | **3.690** | **2.500** |
| *45* | *C123* |  |  | |  | *3.850* | *2.413* |

Training compounds are denoted in bold, while testing compounds are denoted in italics.

**Figure S1.** Williams plot depicting leverage values of the CA IX inhibitors from the library. Only those with leverage values below the threshold (*h**) 0.727 were retained. Values with *h** > 20 have been cut off.

**Statistics of the 2y-GA-PLS model.** Results of cross validation-analysis of variance (CV-ANOVA) used to test the significance of the developed 2y-GA-PLS model (**Table S2**).

**Table S2.** CV-ANOVA results for the **2-y**-GA-PLS model fit to 32 training compounds (α = 0.05).

| GA-PLS | SS | DF | MS | *F* | *p* | SD |
| --- | --- | --- | --- | --- | --- | --- |
| **log*k*_w_** |  |  |  |  |  |  |
| **Total corr.** | 32 | 32 | 1 |  |  | 1 |
| **Regression** | 20.422 | 8 | 2.553 | 5.292 | 0.0007 | 1.598 |
| **Residual** | 11.578 | 24 | 0.482 |  |  | 0.695 |
| **log*K*_i_** |  |  |  |  |  |  |
| **Total corr.** | 32 | 32 | 1 |  |  | 1 |
| **Regression** | 15.872 | 8 | 1.984 | 2.952 | 0.0190 | 1.409 |
| **Residual** | 16.128 | 24 | 0.672 |  |  | 0.820 |

**Figure S2.** Hotelling T^2^ plot for the **2-y**-GA-PLS model; for 33 training compounds.

**Figure S3.** Plot depicting number of simulated compounds against CPU time. CPU time measure using in-built MATLAB function *cputime*.

**Figure S4.** SDS-PAGE gel electrophoresis bands. **A)** CA IX control (2 bands), marker solution (1 band), and CA IX-AZM complexes (9-13 bands), **B)** CA IX-C75, **C)** CA IX-C76, **D)** CA IX-C84, **E)** CA IX-C89, **F)** CA IX-C101, **G)** CA IX-C105, **H)** CA IX-C106.

**Figure S5.** MALDI-TOF/TOF-MS spectra. **A)** CA IX-AZM, **B)** CA IX-C75, **C)** CA IX-C76, **D)** CA IX-C84.

**Figure S6.** FTIR spectra. **A)** CA IX-AZM, **B)** CA IX-C75, **C)** CA IX-C76, **D)** CA IX-C84, **E)** CA IX-C89, **F)** CA IX-C101, **G)** CA IX-C105, **H)** CA IX-C106.

**Figure S7.** Root mean square deviation (RMSD) for CA IX – AZM complex. Fitting with respect to the C-α atoms for CA IX, and the metal site centre.

**Figure S8.** Root mean square deviation (RMSD) for CA IX – C75 complex. Fitting with respect to the C-α atoms for CA IX, and the metal site centre.

**Figure S9.** Root mean square deviation (RMSD) for CA IX –C76 complex. Fitting with respect to the C-α atoms for CA IX, and the metal site centre.

**Figure S10.** Root mean square deviation (RMSD) for CA IX – C89 complex. Fitting with respect to the C-α atoms for CA IX, and the metal site centre.

**Figure S11.** Root mean square deviation (RMSD) for CA IX – C101 complex. Fitting with respect to the C-α atoms for CA IX, and the metal site centre.

**Figure S14.** Distance and angle plots (intermolecular hydrogen bond examination) for CAIX - AZM complex (legend; red: THR200N-THR200H-AM1253O2, olive green: AM1253N3-AM1253H1-THR200OG1).

**Figure S15.** Distance and angle plots (intermolecular hydrogen bond examination) for CAIX – C75 complex (legend; red: S51253N1-S51253H23-THR200OG1, olive green: THR200N-THR200H-S51253O4).

**Figure S16.** Distance and angle plots (intermolecular hydrogen bond examination) for CAIX – C76 complex (legend; red: S61253N1-S61253H23-S61253N11).

**Figure S17.** Distance and angle plots (intermolecular hydrogen bond examination) for CAIX – C89 complex (legend; red: ARG244NH2-ARG244HH21-C91253O15, olive green: C91253N16-C91253H28-GLU106OE1).

**Figure S18.** Distance and angle plots (intermolecular hydrogen bond examination) for CAIX – C101 complex (legend; red: S11253N28-S11253H51-THR200OG1, olive green: THR200N-THR200H-S11253O30, royal blue: S11253N19-S11253H46-GLN71OE1).
